# Old and New Drugs for Chronic Lymphocytic Leukemia: Lights and Shadows of Real-World Evidence

**DOI:** 10.3390/jcm11082076

**Published:** 2022-04-07

**Authors:** Monia Marchetti, Candida Vitale, Gian Matteo Rigolin, Alessandra Vasile, Andrea Visentin, Lydia Scarfò, Marta Coscia, Antonio Cuneo

**Affiliations:** 1 Haematology and Transplant Unit, Azienda Ospedaliera SS Antonio e Biagio e Cesare Arrigo, 15121 Alessandria, Italy; 2 Hematology Unit, Città della Salute e della Scienza, University of Turin, 10126 Turin, Italy; candida.vitale@unito.it (C.V.); marta.coscia@unito.it (M.C.); 3 Haematology Unit, Azienda Ospedaliera Universitaria di Ferrara, 44121 Ferrara, Italy; rglgmt@unife.it (G.M.R.); cut@unife.it (A.C.); 4 Haematology and Rheumatology Section, Department of Medical Sciences, University of Ferrara, 44121 Ferrara, Italy; 5 Department of Public Health, University of Eastern Pedemont, 28100 Novara, Italy; 20034878@studenti.uniupo.it; 6 Hematology and Clinical Immunology Unit, Department of Medicine, University of Padua, 35128 Padua, Italy; andrea.visentin@aopd.veneto.it; 7 Division of Experimental Oncology, Department of Onco-Hematology, IRCCS San Raffaele Hospital, 20132 Milan, Italy; scarfo.lydia@hsr.it

**Keywords:** chronic lymphocytic leukemia, ibrutinib, idelalisib, venetoclax, acalabrutinib, real-world evidence

## Abstract

Several novel treatments for chronic lymphocytic leukemia (CLL) have been recently approved based on the results of randomized clinical trials. However, real-world evidence (RWE) is also requested before and after drug authorization in order to confirm safety and to provide data for health technology assessments. We conducted a scoping review of the available RWE for targeted treatments of CLL, namely ibrutinib, acalabrutinib, idelalisib, and venetoclax, as well as for chemoimmunotherapy (CIT). In particular, we searched studies published since 1 January 2010 and reported outcomes of the above treatments based on health databases, registries, or phase IV studies, including named-patient programs. We included both full papers and abstracts of studies presented at major meetings. Overall, 110 studies were selected and analyzed: 28,880 patients were treated with ibrutinib, 1424 with idelalisib, 751 with venetoclax, 496 with acalabrutinib, and 14,896 with CIT. Reported discontinuation rates were higher than in clinical trials, while effectiveness could not be indirectly compared with clinical trials since a detailed case mix, including cytogenetic risk factors, was partially available and propensity scores rarely applied. RWE on CLL can help to set realistic outcomes with novel treatments, however, real-world studies should be fostered, and available data shared.

## 1. Introduction

Chronic lymphocytic leukemia (CLL) is an indolent lymphoproliferative neoplasm harbored by 5.6 in 10,000 inhabitants (https://seer.cancer.gov/statfacts/html/clyl.html accessed on 30 December 2021). Clinical practice guidelines formerly recommended frontline chemoimmunotherapy (CIT) for all the patients [1,2,3], but several novel treatments, along with several combinations, have been progressively approved in the last 5 years by FDA and EMA: ibrutinib, idelalisib, venetoclax, obinutuzumab, acalabrutinib. The current therapeutic alternatives are, therefore, more diverse than in the past and several concerns apply on patient selection to a personalized treatment sequence. Moreover, patient selection bias due to severe restrictions of patient eligibility to clinical trials is a major hurdle for evidence-based medicine and health technology assessment of novel drugs [4]. Real-world evidence (RWE) gathered either retrospectively or prospectively from electronic health records, medical claims, databases, registries, or patient-generated data can complement the information reported by experimental studies. The pivotal role of RWE is witnessed by the 21st Century Cures Act requiring the FDA to expand the role of RWE and by the European Medicines Agency (EMA) draft guideline on real-world studies (RWS) developed either in the pre- or post-marketing authorization phase of drugs and devices (Table 1). (EMA draft guideline on real world studies 24 September 2020). 

The present scoping review aims at scrutinizing RWE for novel targeted treatments of CLL [5,6], namely ibrutinib, acalabrutinib, idelalisib, and venetoclax, as well as CIT. In particular, we aimed at checking the quality of available registries and named-patient program (NPP)-based studies, patient selection bias (such as age or comorbidities), the quality of available clinical and non-clinical data, and the length of follow-up. 

## 2. Methods

We searched EMBASE, the largest bibliographic database of medical literature, by applying the following mast query: ‘chronic lymphocytic leukemia’ AND (RWD OR ‘real life’ OR ‘real world’ OR EHR OR registry OR register OR registries OR ‘phase IV’ OR post-marketing OR population-based) AND (bendamustine OR fludarabine OR chlorambucil OR venetoclax OR ibrutinib OR idelalisib OR rituximab OR ofatumumab OR obinutuzumab OR acalabrutinib OR rituximab) AND (2010:py OR 2011:py OR 2012:py OR 2013:py OR 2014:py OR 2015:py OR 2016:py OR 2017:py OR 2018:py OR 2019:py OR 2020:py OR 2021:py) AND ‘article’/it. The search was limited to English-language publications reported from 1 January 2010 to 31 December 2020.

Two independent reviewers excluded the inappropriate records and retrieved the data from the selected records. Unspecific reports were deleted. Studies were also excluded if targeted at etiology (familiar lymphoproliferative disorders and exposures), survival, and prognosis (molecular biomarkers and undesirable events). 

The following information were retrieved from selected records: 

Geographic limits

(a) international,
(b) national extensive,
(c) national multi-site,
(d) regional multi-site,
(e) single-institution

Data source

(a) health databases,
(b) existing registry,
(c) newly developed registry,
(d) retrospective chart review,
(e) phase IV study

Population selection

(a) geographic,
(b) treatment,
(c) sample availability,
(d) frailty,
(e) number of lines

Population size

(a) <50,
(b) ≥50

Outcomes

(a) practice patterns,
(b) survival,
(c) prognostic yield of biomarkers,
(d) prognostic yield of a score,
(e) patient-related outcomes (PRO),
(f) health care resource consumption and costs,
(g) adverse events and discontinuation

## 3. Results

**Figure 1:**
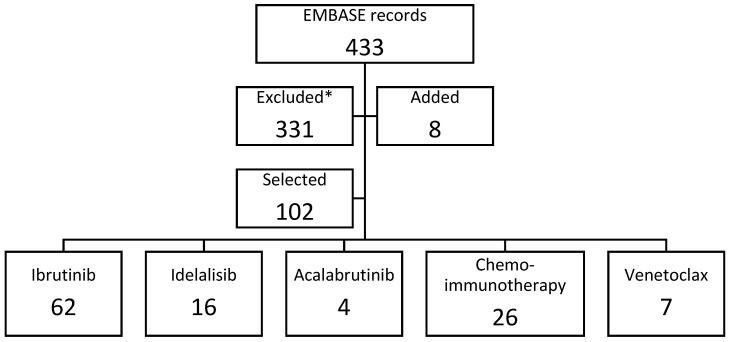
PRISMA diagram.* Reasons for exclusion: less than 20 patients (acalabrutinib), less than 50 patients (other series), mixed treatments, missing outcomes, review.

**Figure 2:**
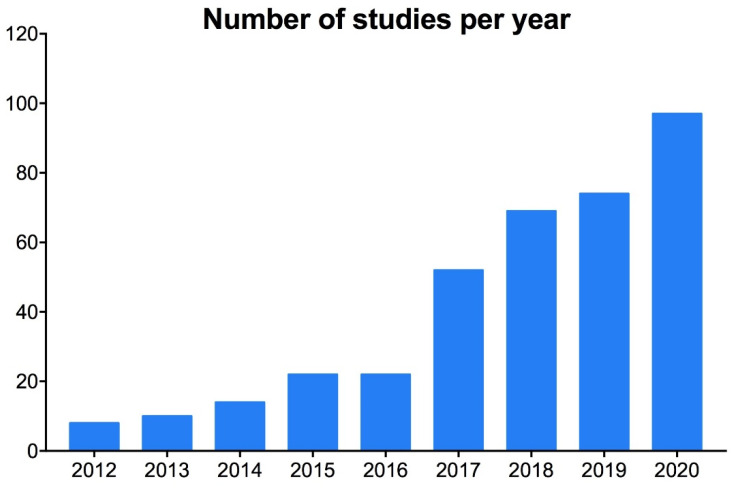
Publication trends of RWS. Distribution of the 433 retrieved RWS according to the year of publication.

Overall, 433 records were retrieved (Figure 1) and showed that the overall trend of publications addressing RWE of CLL rapidly had increased in the last 10 years (Figure 2). The full reference list is reported in Appendix A. Overall, 102 studies were selected from the list and 8 studies were added from the authors, therefore, the search query proved to be quite sensitive. Five studies were retrieved for more than one treatment. The full list of selected RWS is included in Appendix A. Major study characteristics are reported in Table 1. Most of the studies were from single institutions and size was often lower than 500 patients. Patients were representative of the real-life CLL population according to age. However, collected clinical data were unfortunately sparse in most of the published studies, for example, comorbidities were rarely systematically recorded and response rates were not reported by most of the studies. Moreover, follow-ups were short for most of the studies involving new oral inhibitors.

Only 1 phase IV study was reported by Moreno, C. et al. [7]; the study reported the outcomes of 103 patients treated with ofatumumab monotherapy after a median of four prior lines. The study reported a 5-month progression-free survival (PFS) and 11-month overall survival (OS), thus, confirming the data of the published randomized trial comparing ibrutinib versus ofatumumab.

### 3.1. Ibrutinib

Ibrutinib is an orally administered irreversible inhibitor of bruton tyrosine kinase (BTK), representing the first-in-class of a new family of targeted drugs. Ibrutinib covalently binds to cysteine-481 within the active site of BTK, blocking the signal transduction from the B-cell receptor (BCR) and, thus, impairing CLL cells survival and proliferation (reviewed in [8]). Ibrutinib has changed the therapeutic approach for patients with CLL both in first and subsequent lines of therapy, due to impressive response and survival rates, and acceptable tolerability observed in large, randomized clinical trials [9,10,11,12,13].

Many RWS addressed ibrutinib. Overall, 58 nonduplicated reports enrolled more than 50 CLL patients treated with the drug, mostly in the last 10 years. Enrolled populations were potentially representative of the clinical practice: median age was 69 years and median time from diagnosis was 56 months. Unfortunately, only a few studies were powered enough to detect predictors of safety or effectiveness endpoints in a multivariate analysis, namely large registries in the U.S. or healthcare databases. Moreover, both naive and relapsed/refractory patients were usually included. Many pieces of information were missing from many studies: median doses or median treatment durations, response rates, survival rates, and *TP53* mutational status. Several other flaws could be observed in the retrieved studies: limited outcomes were analyzed (i.e., safety or hospitalization rate) and there were no studies that applied propensity-adjusted analyses. Despite the above limitations, the studies provided high discontinuation rates in the short time horizon analyzed. 

### 3.2. Acalabrutinib

Acalabrutinib is a next-generation irreversible BTK inhibitor that was developed to reduce ibrutinib-mediated adverse effects, being more selective and lacking the inhibition towards other kinases (reviewed in [14]). Acalabrutinib has entered clinical practice based on data demonstrating its high efficacy and enhanced tolerability, in both the frontline and relapsed/refractory setting [15].

We retrieved four RWS reporting 496 patients with CLL treated with acalabrutinib; only one RWS was fully reported. A variable preportion of the patients (27–100%) were pretreated with and usually intolerant to ibrutinib. The discontinuation rate reported by two studies ranged from 19% in the first 6 months from treatment initiation to 30% after a median of 19 months. Cardiovascular events occurred in 6% of the largest reported cohort, but led to treatment discontinuation only in half of the cases and corresponded to a rate of 21/1000PY, which was lower than that reported in ibrutinib-treated patients [16].

The overall response rate (ORR) was consistently above 60% in two studies and complete response (CR) was lower than 10%. Survival was documented in two cohorts (median age 64 years); 75% of the patients survived free of progression at three years and 75% were alive after five years, despite a high comorbidity burden, namely mean Charlson comorbidity score 1.4 and 67% of the patients had a prior cardiac history. Patients experiencing a major cardiovascular event reported lower survival rates, namely 50% at 5-year follow-up.

Long-term outcomes such as secondary neoplasms were also investigated in patients exposed to acalabrutinib, however, the increased hazard ratio of 2.2 reported in CLL patients treated with either ibrutinib or acalabrutinib was possibly related to the disease itself, rather than to the treatment. No difference in risk of secondary neoplasms was reported in a multivariate analysis between ibrutinib and acalabrutinib.

A recent retrospective analysis of a large CLL cohort from a single U.S. center specifically investigated the bleeding outcomes [17], however, 85% of the analyzed patients had been enrolled into clinical trials, 18% had prior bleed history, and 51% were on concomitant antiplatelet or anticoagulant medications. Overall, 835 of the patients experienced at least one bleeding event while on acalabrutinib; 98 out of 289 individuals experienced a clinically relevant or major event, but only 6% of the patients had a major bleed and 3% were CTCAE grade 3–5 (2 CNS fatal hemorrhages). Definitive discontinuation of acalabrutinib was decided for 6 patients with clinically relevant/major bleeds, while it was only temporarily held in 44 individuals and concomitant drugs were discontinued in 24 cases. Surgery- or invasive procedure-related bleedings were reported in 28 out of 1263 cases. Concomitant medications and a prior bleeding history were major predictors of bleeding events.

### 3.3. Venetoclax

Venetoclax is an oral BH3-mimetic drug designed to inhibit the function of the Bcl-2 protein, thus, inducing apoptosis in tumor cells (reviewed in [8]). Venetoclax, alone or in combination with an anti-CD20 monoclonal antibody, has demonstrated efficacy for the treatment of patients with treatment-naïve or relapsed CLL, eventually allowing a fixed-duration treatment [18,19,20].

Seven studies reported RWE on venetoclax in CLL patients; one study reported the French national early-access program and the other studies were retrospective national (*n* = 1) or multicenter (*n* = 5) studies. Overall, 751 patients were enrolled into the above studies, most of which were not reported as full papers. Enrolled patients had received a median of from three to four treatment lines, 47% harbored *TP53* mutation, and many showed high-risk features, such as complex karyotype (27–61%) or unmutated IGVH status (81–87%). Grade 3–4 adverse events ranged from 23% to 39% and were mainly due to hematologic toxicity. Discontinuation rate was reported only by two studies and was quite low (4–11%); median treatment duration ranged from 12 to 18 months in two other studies. Response rates were quite high: median ORR was 74% and median CR was 25%. Richter transformation occurred in 4–5% of the patients but was reported only by two studies and the median duration of follow-up was shorter than 20 months in the four studies reporting this piece of information. Median PFS and OS were not reached in any study. Multivariate analyses were performed by five studies: survival was predicted by response to therapy, *TP53* mutation (in two out of three studies), *BCR*-inhibitor discontinuation, multiple lines of target therapies, complex karyotype, performance status, and *IGVH* mutational status. 

### 3.4. Chemoimmunotherapy

Traditionally, CIT was the standard approach in both the frontline and the relapsed/refractory setting of CLL. Common CIT regimens include fludarabine/cyclophosphamide/rituximab (FCR), bendamustine/rituximab (BR), and chlorambucil/rituximab. Older patients or those with comorbidities were recommended to not receive FCR due to the high risk of neutropenic fever and infections, despite very high rates of response in most of the patients [1,2]. More recently, obinutuzumab combination with chlorambucil has progressively replaced chlorambucil/rituximab for the longer median PFS and OS despite similar toxicity, as reported by the CLL14 trial [21,22].

We retrieved 428 studies assessing CIT in real life by health registries (*n* = 13), electronic record databases (*n* = 2), or retrospective data collections (*n* = 11). Most of the studies involved multiple centers (*n* = 15) and followed a median of 277 patients (IQR 174–817) for a median of 37 months (Table 2 and Table 3). The median patient age was 70 years (IQR 64–71) and most of the studies (*n* = 18) selectively enrolled naive individuals, but clinical information was often incomplete (Table 4). In particular, Rai stage and *TP53* status were reported by 64% and 75% of the studies, respectively, and comorbidity data were missing in 61% of the studies. Response rates were reported by only 13 studies: median ORR rate was 61% and complete response (CR) rate 6%. Discontinuation rate was reported by only five studies: median rate was 20%. Patient survival was described by 75% of the studies (Table 3): median PFS and OS among the studies was 42 and 74 months, respectively.

### 3.5. Idelalisib

Idelalisib is an orally administered, selective, reversible inhibitor of the δ isoform of the phosphatidylinositol- 3-kinase (PI3Kδ). The inhibition of *PI3K* downstream pathways (i.e., *Akt*/*mTOR*) hampers cellular growth, proliferation, and survival (reviewed in [23]). From the clinical standpoint, the efficacy of the combination of idelalisib and rituximab has been demonstrated in the setting of relapsed CLL [8].

Overall, 16 studies reported one or more outcomes of real-world cohorts treated with idelalisib; most of the reported patients were relapsed/refractory and more than one third harbored *TP53* disruption. 

The largest cohort was reported by Bird et al. [24]; 294 Medicare patients were compared with 89 patients enrolled into clinical trials. Detailed comorbidity profiles of the patients were provided showing that in the two cohorts, 71% versus 30% of the patients reported cardiac comorbidities, respectively. Similarly, 36% versus 7% showed a Charlson comorbidity score of 5 or higher. Median treatment duration in the Medicare cohort was much shorter than in the trial cohort, namely 173 versus 473 days and on-treatment mortality was 9.9% versus 4.5%. Serious infections were not different in the two cohorts, however, fatal infections occurred at a rate of 18.4/100PY in the Medicare population versus 9.8/100PY in the trial population.

RETRO-idel was the highest quality retrospective study selected; it enrolled 110 patients treated with idelalisib in UK or Ireland, and was fully published in 2021 [25]. The study reported high discontinuation rates both in naiive and relapsed/refractory patients, namely 64% and 44%, respectively, but high ORRs (88%). Overall, 46% of the registered deaths were attributed to progressive disease and OS was 56% after 3 years. Median time-to-next treatment after stopping idelalisib was 29 months.

Two studies were specifically designed to patients reporting autoimmune cytopenias before starting idelalisib or receiving such a therapy as a bridge to stem cell transplantation [26,27]. Only one study reported secondary neoplasms in 12.9% of the patients after a median of 21 months from start of idelalisib treatment; the rates were not statistically different to those reported in ibrutinib-treated patients [28].

## 4. Discussion

CLL is the most frequent leukemia and a large set of modern therapeutic options are available ranging from CIT to oral targeted drugs. These therapeutic options have all reported high rates of responses but also a relevant rate of toxicity in the published clinical trials, which accurately selected candidate patients. We, therefore, aimed at reviewing the available RWE on such therapies in order to test the quality of RWE and to retrieve real-world information for safety and effectiveness. 

EMA specifically fostered the development of patient registries, namely data collection systems on an unselected group of people defined by a particular disease or condition, serving a predetermined scientific, clinical. and/or public health purpose. RWE is particularly relevant for validating safety, including cardiovascular events, and especially for reporting rare events, such as SPM, rare infections, or unexpected events. RWE is also necessary for completing drug effectiveness profiles, including rare events, such as Richter transformation and concurrent disorders. Furthermore, treatment outcomes according to heterogeneous adoption of supportive care are fundamental in order to forecast the overall drug safety in the real world and provide management recommendations [29].

The present manuscript systematically reviewed 117 RWS published from 2010 to 2021 and reported 46,447 CLL patients treated with CIT, idelalisib, ibrutinib, venetoclax or acalabrutinib. Unfortunately, 77 studies had been reported only at meetings and only limited data were available. Most of the studies were multicenter retrospective analyses and most of them targeted only a subset of outcomes. A complete clinical dataset, including age, stage and duration of the disease, comorbidity, and biologic risk status was provided only by a few studies, in particular, stage and disease duration were missing in most of the studies, while *TP53* status was available in 57% of the studies. Only 26% of the RWS reported treatment discontinuation rates and 40% registered response rates; the median response rates ranged from 61% for acalabrutinib to 79% for idelalisib, and CR rate from 6% for acalabrutinib to 30% for CIT. Survival was also poorly described, since median OS or PFS were usually not reached in the follow-up period, which was usually shorter than 2 years in RWS assessing oral target drugs. Of notice, some research networks devoted to CLL published further high-quality RWS both before [30,31] and after the cutoff date of our review [32,33,34,35,36], thus, demonstrating the effort of the scientific community in ameliorating the RWS quality.

The present systematic review aims at fostering further efforts of the scientific community towards RWE, which may become a very useful tool for both researchers and third-party payers. Institutional databases are major prerequisites for RWE, however, further efforts should be aimed at registering response rates, time-to-next treatment, and comorbidities [37]. Finally, large datasets would allow fine analyses including artificial intelligence algorithms and data mining.

## Figures and Tables

**Table 1:** Aims of real-world studies (RWS).

Purposes of RWS before Drug Authorization
To describe the characteristics of the target population
To record the incidence of disease outcomes in the clinical practice
To identify the determinants of disease outcomes in the clinical practice
To provide information on standards of care
**Purposes of RWS after Drug Authorization**
To confirm safety and effectiveness in the target population (i.e., phase IV studies)
To confirm safety in subpopulations (i.e., comorbid patients)
To survey modified patterns of care and health-care resource consumption

**Table 2:** Real-world studies.

	All	Ibrutinib	Idelalisib	Venetoclax	CIT	Acalabrutinib
Study number	117	62	16	7	28 ^	4
Registry	34 (29%)	18	3	0	13	-
NPP/EAP	13 (11%)	10	2	1	-	-
Electronic record database	33 (28%)	28	2	0	2	1
Retrospective data collection	70 (60%)	45	5	6	11	3
Multicenter	52 (44%)	21	10	5	15	1
Europe	40 (45%)	25	11	4	na	-
US/international	41 (46%)	29	5	3	na	4
No explicit patient selection	52 (63%)	37	12	na	na	3
Selectively naiive patients	27 (23%)	8	0	0	18	1
Full papers	40 (34%)	15	5	2	17	1

^ 28 populations from 25 published reports. Legend: NPP, named patient program; EAP, expanded access program; CIT, chemo-immunotherapy; na = not available.

**Table 3:** Information reported by real-world studies.

Studies Reporting:	All	Ibrutinib	Acalabrutinib	Venetoclax	Idelalisib	CIT
Number of treated patients	113 (96%)	58 (93%)	4 (100%)	7 (100%)	16 (100%)	28 (100%)
Patients’ age	90 (77%)	48 (77%)	4 (100%)	5 (71%)	10 (62%)	25 (89%)
Number of treatment lines	61 (52%)	21 (34%)	2 (50%)	5 (71%)	9 (56%)	24 (86%)
Rai/Binet stage	44 (37%)	17 (27%)	1 (25%)	2 (28%)	6 (37%)	18 (64%)
Median time from diagnosis	11 (9%)	8 (13%)	1 (25%)	0	2 (12%)	na
Comorbidity	23 (20%)	7 (11%)	2 (50%)	1 (14%)	2 (12%)	11 (39%)
*TP53* status	67 (57%)	32 (52%)	2 (50%)	5 (71%)	7 (44%)	21 (75%)
Other high-risk molecular or cytogenetic features	61 (52%)	33 (53%)	2 (50%	5 (71%)	2 (12%)	19 (67%)
Median follow-up	78 (66%)	38 (61%)	2 (50%)	4 (57%)	8 (50%)	26 (93%)
Discontinuation rate	31 (26%)	13 (21%)	2 (50%)	2 (28%)	9 (56%)	5 (18%)
Response rate	47 (40%)	20 (32%)	2 (50%)	6 (86%)	6 (37%)	13 (46%)
Richter transformation	10 (8%)	6 (10%)	0	2 (28%)	2 (12%)	na
PFS	52 (44%)	22 (35%)	1 (25%)	4 (57%)	4 (25%)	21 (75%)
OS	61 (52%)	25 (40%)	1 (25%)	5 (71%)	8 (50%)	22 (78%)
TFS or TTNT	16 (14%)	2 (3%)	0	0	2 (12%)	12 (43%)
SPM	12 (10%)	2 (3%)	1 (25%)	0	1 (6%)	8 (28%)
Specific adverse events ^	12 (10%)	4 (6%)	1 (25%)	3 (57%)	4 (25%)	0

^ bleedings and atrial fibrillation for Ibrutinib, cholitis for idelalisib. Legend: CIT, chemoimmunotherapy; SPM, secondary primary malignancies; OS, overall survival; PSF, progression-free survival; TSF, threatment-free survival; TTNT, time-to-next treatment; na = not available.

**Table 4:** Patient characteristics.

	Ibrutinib	Idelalisib	Venetoclax	CIT	Acalabrutinib
Patient number: Mean, median, inter-quartile range	486 179 89–554	89 74 54–104	107 76 67–149	532 277 174–817	149 136 33–290
Age: Median years, inter-quartile range	69 65–70	72 67–74	68 67–69	70 68–75	68 64–71
Number of prior treatment lines: Median, inter-quartile range	2 0–3	3 1–4	3.5 3–4	0 0–1	4 (1 study)
Follow-up (mo): Median, inter-quartile range	16 9–21	16 7–19	13 9–17	37 25–57	12 5–19
Discontinuation rate: Range	23–41%	63–100%	7–27%	2–30%	19–30%
Overall Response Rate: Median, inter-quartile range	77% 73–84%	79% 65–86%	74% 72–75%	83% 76–91%	61% 60–62%
Complete Response Rate: Median, Inter-quartile range	17% 11–18%	14% 1 study	25% 23–28%	30% 19–55%	6% 3–9%
PFS: Median, range,	38 na	na 10–36	na na	42 28–51	na na
OS	na *	>36	na	74	na

* median OS was usually not reached in the median follow-up reported. Median reported values were usually >48 months for first-line treatment. na = not available.

## Appendix A

**Table A1:** References selected from embase query.

Author Names	Title	Source
Aarup K., Enggaard L., Pedersen R.S., Thomsen R.H., Bergmann O.J., Frederiksen M., Christiansen I., Nielsen T., Frederiksen H., Niemann C.U., Andersen M.A.	Real-world outcomes for 205 Danish patients with chronic lymphocytic leukemia treated with ibrutinib	Blood (2019) 134 Supplement 1. Date of Publication: 1 Nov 2019
Aarup K., Rotbain E.C., Enggaard L., Pedersen R.S., Bergmann O.J., Thomsen R.H., Frederiksen M., Frederiksen H., Nielsen T., Christiansen I., Andersen M.A., Niemann C.U.	Real-world outcomes for 205 patients with chronic lymphocytic leukemia treated with ibrutinib	*European Journal of Haematology* (2020) 105:5 (646–654). Date of Publication: 1 Nov 2020
Abrisqueta Costa D.P., Loscertales D.J., Terol D.M.J., Ramírez Payer D.Á., Ortiz D.M., Pérez D.I., Moreno D.C., Fernández De La Mata D.M., Rodríguez D.A., Lario D.A., Delgado D.J., Godoy D.A., Arguiñano Pérez D.J.M., Berruezo D.M.J., Oliveira D.A., Hernández Rivas D.J.Á., García D.L., Medina D.Á., García Martin D.P., Osorio D.S., Baltasar D.P., Fernández D.M., Marco D.F., Vidal Manceñido D.M.J., Smucler Simonovich A., López Rubio M., Jarque D.I., Suarez D.A., Fernández Álvarez D.R., Lancharro Anchel D.A., Ríos D.E., Losada Castillo D.M.D.C., Pérez Persona D.E., García Muñoz D.R., Ramos D.R., Yáñez D.L., Luis Bello D.J., Villanueva D.M.	Retrospective observational study of the treatment of chronic lymphocytic leukemia (CLL) with ibrutinib in routine clinical practice in spain	*HemaSphere* (2020) 4 Supplement 1 (311–312). Date of Publication: 1 Jun 2020
Akhtar O.S., Attwood K., Lund I., Hare R., Hernandez-Ilizaliturri F.J., Torka P.	Dose reductions in ibrutinib therapy are not associated with inferior outcomes in patients with chronic lymphocytic leukemia (CLL)	*Leukemia and Lymphoma* (2019) 60:7 (1650–1655). Date of Publication: 7 Jun 2019
Akhtar O.S., Torka P., Bhat S.A., Hare R., Sait S.N.J., Block A.W., Hernandez-Ilizaliturri F.J.	Disease progression on ibrutinib therapy is associated with a poor clinical outcome in chronic lymphocytic leukemia (CLL) patients managed in standard clinical practice	*Blood* (2017) 130 Supplement 1. Date of Publication: 1 Dec 2017
Allouchery M., Delaunay P., Fourrier-Réglat A., Lafay-Chebassier C., Pérault-Pochat M.C.	Patterns of use and safety of ibrutinib in a real life practice: Preliminary results of an observational cohort study	*Fundamental and Clinical Pharmacology* (2019) 33 Supplement 1 (6). Date of Publication: 1 Jun 2019
Autore F., Innocenti I., Corrente F., Del Principe M.I., Rosati S., Falcucci P., Fresa A., Conte E., Limongiello M.A., Renzi D., De Padua L., Andriani A., Pisani F., Cimino G., Tafuri A., Montanaro M., Mauro F.R., Del Poeta G., Laurenti L.	Front-Line Therapy for Elderly Chronic Lymphocytic Leukemia Patients: Bendamustine Plus Rituximab or Chlorambucil Plus Rituximab? Real-Life Retrospective Multicenter Study in the Lazio Region	*Frontiers in Oncology* (2020) 10 Article Number: 848. Date of Publication: 10 Jun 2020
Beiggi S., Banerji V., Deneka A., Griffith J., Gibson S.B., Johnston J.B.	Comparison of outcome of patients with CLL who are referred or nonreferred to a specialized CLL clinic: a Canadian population-based study	*Cancer Medicine* (2016) 5:6 (971–979). Date of Publication: 1 Jun 2016
Bird S.T., Tian F., Flowers N., Przepiorka D., Wang R., Jung T.-H., Kessler Z., Woods C., Kim B., Miller B.W., Wernecke M., Kim C., McKean S., Gelperin K., MacUrdy T.E., Kelman J.A., Graham D.J.	Idelalisib for Treatment of Relapsed Follicular Lymphoma and Chronic Lymphocytic Leukemia: A Comparison of Treatment Outcomes in Clinical Trial Participants vs Medicare Beneficiaries	*JAMA Oncology* (2020) 6:2 (248–254). Date of Publication: 1 Feb 2020
Bouclet F., Calleja A., Dilhuydy M.-S., Amorim S., Cymbalista F., Herbaux C., De Guibert S., Roos-Weil D., Hivert B., Aurran T., Dupuis J., Blouet A., Tchernonog E., Laribi K., Dmytruk N., Morel P., Michallet A.-S., Dartigeas C., Farnault L., Lavaud A., Plantier I., Bay J.-O., Tournilhac O., Delmer A.J., Feugier P., Ysebaert L., Guieze R.	Outcome of patients receiving venetoclax for chronic lymphocytic leukemia (CLL) in real-life clinical practice: Results of the French ATU program on behalf of the Filo Group	*Blood* (2018) 132 Suppl. 1. Date of Publication: 1 Nov 2018
Brander D.M., Rhodes J., Pagel J.M., Nabhan C., Tam C.S., Jacobs R., Hill B.T., Lamanna N., Lansigan F., Shadman M., Ujjani C.S., Skarbnik A.P., Cheson B.D., Pu J.J., Sehgal A.R., Barr P.M., Allan J.N., Beach D.F., Patel B., Pickens P.V., Nasta S.D., Kennard K., Tuncer H.H., Koch B., Furman R.R., Mato A.R.	Applicability of the chronic lymphocytic leukemia (CLL)-IPI on patients treated with front-line ibrutinib in the real world: The case for new prognostic models	*Blood* (2017) 130 Supplement 1. Date of Publication: 1 Dec 2017
Cassin R., Visentin A., Noto A., Giannarelli D., Mauro F.R., Baldini L., Trentin L., Reda G.	Hypogammaglobulinaemia and infections in ibrutinib treated chronic lymphocytic leukemia (CLL) pazients: A prospective study	*HemaSphere* (2020) 4 Supplement 1 (308–309). Date of Publication: 1 Jun 2020
Chien H.-C., Patil V., Rasmussen K.M., Yong C., Biondo J.M.L., Halloran M., Shapouri S., Wu M., Burningham Z.R., Sauer B.C., Halwani A.S.	Discontinuation patterns in patients receiving novel oral agents for chronic lymphocytic leukemia in the veterans health administration	*Blood* (2019) 134 Supplement 1. Date of Publication: 1 Nov 2019
Cuneo A., Follows G., Rigolin G.M., Piciocchi A., Tedeschi A., Trentin L., Perez A.M., Coscia M., Laurenti L., Musuraca G., Farina L., Delgado A.R., Orlandi E.M., Galieni P., Mauro F.R., Visco C., Amendola A., Billio A., Marasca R., Chiarenza A., Meneghini V., Ilariucci F., Marchetti M., Molica S., Re F., Gaidano G., Gonzalez M., Forconi F., Ciolli S., Cortelezzi A., Montillo M., Smolej L., Schuh A., Eyre T.A., Kennedy B., Bowles K.M., Vignetti M., De La Serna J., Moreno C., Foà R., Ghia P.	Efficacy of bendamustine and rituximab as first salvage treatment in chronic lymphocytic leukemia and indirect comparison with ibrutinib: A GIMEMA, ERIC and UK CLL FORUM study	Haematologica (2018) 103:7 (1209–1217). Date of Publication: 3 Jul 2018
Cuneo A., Mato A.R., Rigolin G.M., Piciocchi A., Gentile M., Laurenti L., Allan J.N., Pagel J.M., Brander D.M., Hill B.T., Winter A., Lamanna N., Tam C.S., Jacobs R., Lansigan F., Barr P.M., Shadman M., Skarbnik A.P., Pu J.J., Sehgal A.R., Schuster S.J., Shah N.N., Ujjani C.S., Roeker L., Orlandi E.M., Billio A., Trentin L., Spacek M., Marchetti M., Tedeschi A., Ilariucci F., Gaidano G., Doubek M., Farina L., Molica S., Di Raimondo F., Coscia M., Mauro F.R., de la Serna J., Medina Perez A., Ferrarini I., Cimino G., Cavallari M., Cucci R., Vignetti M., Foà R., Ghia P.	Efficacy of bendamustine and rituximab in unfit patients with previously untreated chronic lymphocytic leukemia. Indirect comparison with ibrutinib in a real-world setting: A GIMEMA-ERIC and US study	*Cancer Medicine* (2020) 9:22 (8468–8479). Date of Publication: 1 Nov 2020
DaCosta Byfield S., Blauer-Peterson C., Dawson K., Masaquel A.	What are the health care utilization and costs associated with patients newly initiating anti-cancer systemic therapy for chronic lymphocytic leukemia?	*Journal of Managed Care and Specialty Pharmacy* (2018) 24 4-A SUPPL. (S29). Date of Publication: 1 Apr 2018
Dartigeas C., Feugier P., Ysebaert L., Dilhuydy M.-S., Delmer A., Tardy S., Anglaret B., Voillat L., Le Dû K., Slama B., Albrecht C., Jenayah L., Beauclair S., Wapenaar R., Kavanagh C., Leblond V.	French ibrutinib observational study (FIRE): Real-world study of ibrutinib treatment for chronic lymphocytic leukemia (CLL) in france	*HemaSphere* (2019) 3 Supplement 1 (145–146). Date of Publication: 1 Jun 2019
Del Poeta G., Biagi A., Laurenti L., Chiarenza A., Pozzo F., Innocenti I., Postorino M., Rossi F.M., Del Principe M.I., Bomben R., de Fabritiis P., Bruno A., Cantonetti M., Di Raimondo F., Zucchetto A., Gattei V.	Impaired nodal shrinkage and apoptosis define the independent adverse outcome of NOTCH1 mutated patients under ibrutinib therapy in chronic lymphocytic leukaemia	*Haematologica* (2020). Date of Publication: 30 Jul 2020
Dimou M., Iliakis T., Pardalis V., Bitsani C., Vassilakopoulos T.P., Angelopoulou M., Tsaftaridis P., Papaioannou P., Koudouna A., Kalyva S., Kyrtsonis M.-C., Panayiotidis P.	Safety and efficacy analysis of long-term follow up real-world data with ibrutinib monotherapy in 58 patients with CLL treated in a single-center in Greece	*Leukemia and Lymphoma* (2019) 60:12 (2939–2945). Date of Publication: 15 Oct 2019
Diop F., Moia R., Favini C., Spaccarotella E., De Paoli L., Bruscaggin A., Spina V., Cerri M., Deambrogi C., Kodipad A.A., Favini S., Sagiraju S., Jabangwe C., Mauro F.R., Del Giudice I., Forconi F., Cortelezzi A., Zaja F., Visco C., Chiarenza A., Rigolin G.M., Marasca R., Coscia M., Perbellini O., Tedeschi A., Laurenti L., Motta M., Del Poeta G., Cuneo A., Gattei V., Foa R., Gaidano G., Rossi D.	BRAF and BIRC3 mutations stratify a poor prognostic subgroup in FCR treated chronic lymphocytic leukemia	*Blood* (2017) 130 Supplement 1. Date of Publication: 1 Dec 2017
Emole J.N., Viganego F., Schabath M.B., Shah B.D., Chavez J.C., Walko C.M., McLeod H.L., Pinilla-Ibarz J., Fradley M.G.	Incidence and risk factors for ibrutinib associated atrial fibrillation	*Journal of Clinical Oncology* (2016) 34 Supplement 15. Date of Publication: 1 May 2016
Ferra C.M., Encinas M.P., Jimenez J.L., Ortiz M., Osorio-Prendes S., Cordoba R., Payer A.R., González-Barca E., Sánchez G.M., Diaz M.G., Sanchez M.J., Fernandez M., Tello P.B., Amutio E., García-Malo M.-D., MacEñido M.J.V., Fernandez P., Loscertales J., Rodríguez J.N., Alaez C., Ramroth H., Palla M.	Retrospective non-interventional assessment of the use of idelalisib in relapsed/refractory chronic lymphocytic leukemia patients in Spain	Blood (2019) 134 Supplement 1. Date of Publication: 1 Nov 2019
Finnes H.D., Chaffee K.G., Call T.G., Ding W., Bowen D.A., Conte M., McCullough K.B., Merten J.A., Bartoo G.T., Smith M.D., Schwager S.M., Slager S.L., Kay N.E., Shanafelt T.D., Parikh S.A.	The importance of pharmacovigilance during ibrutinib therapy for chronic lymphocytic leukemia (CLL) in routine clinical practice	*Blood* (2015) 126:23 (717). Date of Publication: 3 Dec 2015
Frei M., Aitken S.L., Jain N., Thompson P., Wierda W.G., Kontoyiannis D.P., DiPippo A.	Incidence and characterization of invasive fungal infections (IFIS) in patients with chronic lymphocytic leukemia (CLL) treated with ibrutinib (IBR)	*Open Forum Infectious Diseases* (2019) 6 Supplement 2 (S630). Date of Publication: 1 Oct 2019
Gentile M., Morabito F., Del Poeta G., Mauro F.R., Reda G., Sportoletti P., Laurenti L., Coscia M., Herishanu Y., Recchia A.G., Varettoni M., Murru R., Chiarenza A., Condoluci A., Moia R., Pietrasanta D., Loseto G., Consoli U., Scortechini I., Rossi F.M., Zucchetto A., Fraticelli V., Vigna E., Botta C., Tripepi G., Arrigo G.D., Rago A., Angeletti I., Biagi A., Del Giudice I., Bomben R., Rigolin G.M., Rossi D., Di Raimondo F., Gaidano G., Polliack A., Cuneo A., Foà R., Gattei V.	Survival risk score for real-life relapsed/refractory chronic lymphocytic leukemia patients receiving ibrutinib. A campus CLL study	*Leukemia* (2020). Date of Publication: 2020
Gentile M., Morabito F., Del Poeta G., Mauro F.R., Reda G., Sportoletti P., Laurenti L., Coscia M., Herishanu Y., Recchia A.G., Varettoni M., Murru R., Chiarenza A., Condoluci A., Moia R., Pietrasanta D., Loseto G., Consoli U., Scortechini I., Rossi F.M., Zucchetto A., Vigna E., Tripepi G., Rago A., Angeletti I., Biagi A., Del Giudice I., Bomben R., Rigolin G.M., Rossi D., Di Raimondo F., Gaidano G., Polliack A., Cuneo A., Foà R., Gattei V.	External validation of a novel risk model (BALL Score) in real-world relapsed/refractory chronic lymphocytic leukemia patients receiving ibrutinib: a campus cll study	*Blood* (2019) 134 Supplement 1. Date of Publication: 1 Nov 2019
Georgantopoulos P., Yang H., Norris L.B., Bennett C.L.	Major hemorrhage in chronic lymphocytic leukemia patients in the U.S.Veterans Health Administration system in the pre-ibrutinib era: Incidence and risk factors	*Cancer Medicine* (2019) 8:5 (2233–2240). Date of Publication: 1 May 2019
Goyal R.K., Nagar S.P., Kabadi S.M., Davis K.L., Le H., Kaye J.A.	Overall survival adverse events, and economic burden in medicare patients with chronic lymphocytic leukemia receiving cancer-directed therapy	*Blood* (2019) 134 Supplement 1. Date of Publication: 1 Nov 2019
Halwani A.S., Rasmussen K., Patil V., Burningham Z., Sauer B.C.	Incidence of atrial fibrillation and bleeding in CLL patients treated with ibrutinib: Evidence from the veterans health administration	*Blood* (2017) 130 Supplement 1. Date of Publication: 1 Dec 2017
Hansson L., Winqvist M., Asklid A., Andersson P.-O., Karlsson K., Karlsson C., Lauri B., Lundin J., Mattsson M., Norin S., Sandstedt A., Österborg A.	Real-world results on ibrutinib in patients with relapsed or refractory chronic lymphocytic leukemia (CLL): Data from 97 swedish patients treated in a compassionate use program	*Blood* (2015) 126:23 (1745). Date of Publication: 3 Dec 2015
Herishanu Y., Goldschmidt N., Bairey O., Ruchlemer R., Fineman R., Rahimi-Levene N., Shvidel L., Tadmor T., Ariel A., Braester A., Shapiro M., Joffe E., Polliack A.	Efficacy and safety of front-line therapy with fludarabine-cyclophosphamide-rituximab regimen for chronic lymphocytic leukemia outside clinical trials: The Israeli CLL study group experience	*Haematologica* (2015) 100:5 (662–669). Date of Publication: 2015
Herishanu Y., Shaulov A., Fineman R., Bašić-Kinda S., Aviv A., Wasik-Szczepanek E., Jaksic O., Zdrenghea M., Greenbaum U., Mandac I., Simkovic M., Morawska M., Benjamini O., Spacek M., Nemets A., Bairey O., Trentin L., Ruchlemer R., Laurenti L., Stanca Ciocan O., Doubek M., Shvidel L., Dali N., Mirás F., De Meûter A., Dimou M., Mauro F.R., Coscia M., Bumbea H., Szász R., Tadmor T., Gutwein O., Gentile M., Scarfò L., Tedeschi A., Sportoletti P., Gimeno Vázquez E., Marquet J., Assouline S., Papaioannou M., Braester A., Levato L., Gregor M., Rigolin G.M., Loscertales J., Medina Perez A., Nijziel M.R., Popov V.M., Collado R., Slavutsky I., Itchaki G., Ringelstein S., Goldschmidt N., Perry C., Levi S., Polliack A., Ghia P.	Frontline treatment with the combination obinutuzumab ± chlorambucil for chronic lymphocytic leukemia outside clinical trials: Results of a multinational, multicenter study by ERIC and the Israeli CLL study group	*American Journal of Hematology* (2020) 95:6 (604–611). Date of Publication: 1 Jun 2020
Hillmen P., Diels J., Healy N., Iraqi W., Aschan J., Wildgust M.	Real-world experience of ibrutinib in >2900 chronic lymphocytic leukemia patients: Data from a global named patient program	*Haematologica* (2016) 101 Supplement 1 (51). Date of Publication: 1 Jun 2016
Huang Q., Borra S., Li J., Wang L., Shrestha S., Sundaram M., Janjan N.	Time to next treatment, health care resource utilization, and costs associated with ibrutinib use among u.s. veterans with chronic lymphocytic leukemia/small lymphocytic lymphoma: A real-world retrospective analysis	*Journal of Managed Care and Specialty Pharmacy* (2020) 26:10 (1266–1275). Date of Publication: 1 Oct 2020
Huang Q., Ellis L., Wang L., Shrestha S., Sundaram M.	Real-world evidence of ibrutinib use among patients with chronic lymphocytic leukemia and/or small lymphocytic lymphoma in the u.s. veterans health administration population	*Journal of Managed Care and Specialty Pharmacy* (2019) 25 10-A SUPPL. (S36). Date of Publication: 1 Oct 2019
Huang Q., Ellis L., Wang L., Shrestha S., Sundaram M.	First-line therapy in chronic lymphocytic leukemia: A Swedish nation-wide real-world study on 1053 consecutive patients treated between 2007 and 2013	*Clinical Lymphoma, Myeloma and Leukemia* (2019) 19 Supplement 1 (S285). Date of Publication: 1 Sep 2019
Huang Q., Emond B., Lafeuille M.H., Gupta D., Lefebvre P., Sundaram M., Mato A.	PCN124 real-world healthcare resource utilization and total cost of care among us medicare patients with chronic lymphocytic leukemia receiving first-line ibrutinib vs. chemoimmunotherapy	*Value in Health* (2020) 23 Supplement 1 (S44-S45). Date of Publication: 1 May 2020
Huang S.J., Lee L.J., Gerrie A.S., Gillan T.L., Bruyere H., Hrynchak M., Smith A.C., Karsan A., Ramadan K.M., Jayasundara K.S., Toze C.L.	Characterization of treatment and outcomes in a population-based cohort of patients with chronic lymphocytic leukemia referred for cytogenetic testing in British Columbia, Canada	*Leukemia Research* (2017) 55 (79–90). Date of Publication: 1 Apr 2017
Huntington S.F., Soulos P.R., Barr P.M., Jacobs R., Lansigan F., Odejide O.O., Schwartzberg L.S., Davidoff A.J., Gross C.P.	Utilization and early discontinuation of first-line ibrutinib for patients with chronic lymphocytic leukemia treated in the community oncology setting in the United States	*Blood* (2019) 134 Supplement 1. Date of Publication: 1 Nov 2019
Irwin D., Szabo E., Pathak A., Tang B.	Length of stay for hospitalized patients treated with ibrutinib or bendamustine first-line therapy for treatment of chronic lymphocytic leukemia	*Blood* (2017) 130 Supplement 1. Date of Publication: 1 Dec 2017
Irwin D., Wilson K.	Characteristics and treatment patterns of patients receiving oral ibrutinib therapy for treatment of chronic lymphocytic leukemia	*Journal of Managed Care and Specialty Pharmacy* (2017) 23 3-A SUPPL. (S34). Date of Publication: 1 Mar 2017
Iskierka-Jazdzewska E., Hus M., Giannopoulos K., Madro E., Hołojda J., Piotrowska M., Zaucha J.M., Piszczek W., Szeremet A., Wojciechowska M., Steckiewicz P., Knopińska-Posłuszny W., Osowiecki M., Drozd-Sokołowska J., Kumiega B., Kyrcz-Krzemień S., Hałka J., Dudziński M., Wieszczy P., Warzocha K., Jamroziak K.	Results of a prospective observational trial of polish adult leukemia group (PALG) on ibrutinib compassionate use in relapsed or refractory chronic lymphocytic leukaemia (CLL) in poland	*Haematologica* (2016) 101 Supplement 1 (439–440). Date of Publication: 1 Jun 2016
Iyengar R., Gutierrez M., Ghosh N., Barrientos J., Brander D., Kadish K., Tomlinson B., Mato A., Sharman J., Ipe D., Han J., Amaya-Chanaga C., Sundaram M.	Treatment patterns, clinical outcomes, and healthcare resource utilization for previously untreated and relapsed/ refractory patients with chronic lymphocytic leukemia in the era of novel agents: Interim analysis from the informcll registry	*Journal of Managed Care and Specialty Pharmacy* (2019) 25 10-A SUPPL. (S35). Date of Publication: 1 Oct 2019
Iyengar R., Malangone-Monaco E., Sugg C., Amaya-Chanaga C., McMorrow D., Shukrun N., Balakrishnan C., Giafis N.	Comparison of healthcare resource utilization and total direct costs for chronic lymphocytic leukemia patients treated with ibrutinib or chemoimmunotherapy	*Journal of Managed Care and Specialty Pharmacy* (2019) 25 10-A SUPPL. (S36). Date of Publication: 1 Oct 2019
Janssens A., André M., Berneman Z., Snauwaert S., De Beleyr B., Smet A., Van Bogaert C., Wapenaar R., Bron D.	Effectiveness and safety of ibrutinib for chronic lymphocytic leukemia (CLL) in routine clinical practice: Interim analysis (IA) of the belgian ibrutinib real-world data (BiRD) study	*HemaSphere* (2019) 3 Supplement 1 (144). Date of Publication: 1 Jun 2019
Kleeberg U.R., Linde H., Günther G., Tessen H.-W., Kersting M.	Bendamustin-rituximab combination is a safe and effective, ambulatory treatment for elderly patients with chronic lymphocytic leukemia: Retrospective real-world analysis by age from a German registry and review of the literature	*Anticancer Research* (2016) 36:6 (2827–2838). Date of Publication: 1 Jun 2016
Knauf W., Hoechstetter M., Eissmann P., Hucke N., Van Troostenburg A., Ramroth H., Rummel M.	Prospective real world data of an ongoing post-authorization safety study on idelalisib in patients with CLL and refractory FL	*HemaSphere* (2018) 2 Supplement 2 (852–853). Date of Publication: 1 Jun 2018
Kunk P.R., Mock J., Devitt M.E., Palkimas S., Sen J., Portell C.A., Williams M.E.	Major bleeding with ibrutinib: More than expected	*Blood* (2016) 128:22. Date of Publication: 2 Dec 2016
Kuranz S., Stacey J., Luciano S.	Early discontinuation (ED) of therapy and treatment patterns among chronic lymphocytic leukemia (CLL) patients: Findings from a linked claims and electronic health record (EHR) dataset	*Blood* (2019) 134 Supplement 1. Date of Publication: 1 Nov 2019
Lee L.J., Toze C.L., Huang S.J.T., Gillan T.L., Connors J.M., Sehn L.H., Bruyere H., Leitch H., Ramadan K.M., Gerrie A.S.	Improved survival outcomes with the addition of rituximab to initial therapy for chronic lymphocytic leukemia: a comparative effectiveness analysis in the province of British Columbia, Canada	*Leukemia and Lymphoma* (2018) 59:6 (1356–1363). Date of Publication: 3 Jun 2018
Lenartova A., Johannesen T.B., Tjønnfjord G.E.	Chronic lymphocytic leukemia and secondary hematological malignancies: A nation-wide cancer registry study	*European Journal of Haematology* (2020) 104:6 (546–553). Date of Publication: 1 Jun 2020
Li J.J., Yong A.S., Justicia J.L., Smith C., Delgado J.	Idelalisib in combination with rituximab in chronic lymphocytic leukemia (CLL)/small lymphocytic lymphoma (SLL): Real-world experience through an early access program in Europe and Australia	*Haematologica* (2016) 101 Supplement 1 (229). Date of Publication: 1 Jun 2016
Mato A., Nabhan C., Lamanna N., Kay N.E., Grinblatt D.L., Flowers C.R., Farber C.M., Davids M.S., Swern A.S., Sullivan K., Dawn Flick E., Gressett Ussery S.M., Gharibo M., Kiselev P., Sharman J.P.	The Connect CLL Registry: Final analysis of 1494 patients with chronic lymphocytic leukemia across 199 US sites	*Blood Advances* (2020) 4:7 (1407–1418). Date of Publication: 14 Apr 2020
Mato A.R., Allan J.N., Pagel J.M., Brander D.M., Hill B.T., Cheson B.D., Furman R.R., Lamanna N., Tam C.S., Jacobs R., Lansigan F., Barr P.M., Shadman M., Skarbnik A.P., Beach D.F., Pu J.J., Sehgal A.R., Winter A.M., Zent C.S., Tuncer H.H., Singavi A.K., Schuster S.J., Pickens P.V., Shah N.N., Williams A., Howlett C., Weissbrot H., Ali N., Patel B., Isaac K., Rhodes J., Hughes M.E., Khajavian S., Chatburn E.T., Sitlinger A., Tranchito E., Bhavsar E.B., Bailey N., Burns T.F., Yacur M., Malhotra M., Handunnetti S., Kennard K., Nabhan C., Ujjani C.S.	Front-line ibrutinib therapy for chronic lymphocytic leukemia (CLL) in the real world: Responses, toxicity, outcomes, and subsequent therapies	*Blood* (2017) 130 Supplement 1. Date of Publication: 1 Dec 2017
Mato A.R., Barrientos J.C., Ghosh N., Pagel J.M., Brander D.M., Gutierrez M., Kadish K., Tomlinson B., Iyengar R., Ipe D., Upasani S., Amaya-Chanaga C.I., Sundaram M., Han J., Giafis N., Sharman J.P.	Prognostic Testing and Treatment Patterns in Chronic Lymphocytic Leukemia in the Era of Novel Targeted Therapies: Results From the informCLL Registry	*Clinical Lymphoma, Myeloma and Leukemia* (2020) 20:3 (174–183.e3). Date of Publication: 1 Mar 2020
Mato A.R., Hill B.T., Lamanna N., Barr P.M., Ujjani C.S., Brander D.M., Howlett C., Skarbnik A.P., Cheson B.D., Zent C.S., Pu J.J., Kiselev P., Foon K., Lenhart J., Henick Bachow S., Winter A.M., Cruz A.-L., Claxton D.F., Goy A., Daniel C., Isaac K., Kennard K.H., Timlin C., Fanning M., Gashonia L., Yacur M., Svoboda J., Schuster S.J., Nabhan C.	Optimal sequencing of ibrutinib, idelalisib, and venetoclax in chronic lymphocytic leukemia: results from a multicenter study of 683 patients	*Annals of oncology: official journal of the European Society for Medical Oncology* (2017) 28:5 (1050–1056). Date of Publication: 1 May 2017
Mato A.R., Nabhan C., Barr P.M., Ujjani C.S., Hill B.T., Lamanna N., Skarbnik A.P., Howlett C., Pu J.J., Sehgal A.R., Strelec L.E., Vandegrift A., Fitzpatrick D.M., Zent C.S., Feldman T., Goy A., Claxton D.F., Bachow S.H., Kaur G., Svoboda J., Nasta S.D., Porter D., Landsburg D.J., Schuster S.J., Cheson B.D., Kiselev P., Evens A.M.	Outcomes of CLL patients treated with sequential kinase inhibitor therapy: A real-world experience	*Blood* (2016) 128:18 (2199–2205). Date of Publication: 3 Nov 2016
Mato A.R., Nabhan C., Thompson M.C., Lamanna N., Brander D.M., Hill B., Howlett C., Skarbnik A., Cheson B.D., Zent C., Pu J., Kiselev P., Goy A., Claxton D., Isaac K., Kennard K.H., Timlin C., Landsburg D., Winter A., Nasta S.D., Bachow S.H., Schuster S.J., Dorsey C., Svoboda J., Barr P., Ujjani C.S.	Toxicities and outcomes of 616 ibrutinib-treated patients in the united states: A real-world analysis	*Haematologica* (2018) 103:5 (874–879). Date of Publication: 30 Apr 2018
Mato A.R., Sail K., Yazdy M.S., Hill B.T., Shadman M., Manzoor B.S., Tuncer H.H., Allan J.N., Ujjani C.S., Sharmokh S., Jiang D.D., Pena G., Marshall T., Nielsen J., Barr P.M., Brown J.R., Schuh A., Eyre T.A., Wierda W.G., Skarbnik A., Roeker L.E., Bannerji R., Pauff J.M., Schuster S.J., Follows G.A., Cheson B.D., Eichhorst B.F., Brander D.M., Pivneva I., Lamanna N.	Treatment sequences and outcomes of patients with CLL treated with venetoclax and other novel agents post introduction of novel therapies	*Blood* (2019) 134 Supplement 1. Date of Publication: 1 Nov 2019
Mato A.R., Thompson M., Allan J.N., Brander D.M., Pagel J.M., Ujjani C.S., Hill B.T., Lamanna N., Lansigan F., Jacobs R., Shadman M., Skarbnik A.P., Pu J.J., Barr P.M., Sehgal A.R., Cheson B.D., Zent C.S., Tuncer H.H., Schuster S.J., Pickens P.V., Shah N.N., Goy A., Winter A.M., Garcia C., Kennard K., Isaac K., Dorsey C., Gashonia L.M., Singavi A.K., Roeker L.E., Zelenetz A., Williams A., Howlett C., Weissbrot H., Ali N., Khajavian S., Sitlinger A., Tranchito E., Rhodes J., Felsenfeld J., Bailey N., Patel B., Burns T.F., Yacur M., Malhotra M., Svoboda J., Furman R.R., Nabhan C.	Real-world outcomes and management strategies for venetoclax-treated chronic lymphocytic leukemia patients in the United States	*Haematologica* (2018) 103:9 (1511–1517). Date of Publication: 31 Aug 2018
Mauro F.R., Giannarelli D., Visentin A., Reda G., Coscia M., Tedeschi A., Sportoletti P., Chiarenza A., Ciolli S., Levato L., Gentile M., Laurenti L., Rigolin G.M., Vitale C., Giordano A.M., Murru R., De Paoli L., Del Poeta G., Rosati S., Riemma C., Cassin R., Frustaci A.M., Stelitano C., Cuneo A., Molica S., Girmenia C., Foà R., Trentin L.	Severe infections and pneumonia in patients with chronic lymphocytic leukemia (CLL) treated with Kinase inhibitors (KIS) ibrutinib or idelalisib in the real world	*Haematologica* (2019) 104 Supplement 2 (100). Date of Publication: 1 Oct 2019
Mauro F.R., Tedeschi A., Piciocchi A., Motta M., Iannella E., Farina L., Scarfo L., Marasca R., Coscia M., Cortelezzi A., Laurenti L., Melpignano A., Zinzani P.L., Molica S., Re F., Andriani A., Vincelli D.I., Visco C., Gozzetti A., Orlandi E.M., Trentin L., Tani M., Califano C., Tagariello G., Ghia P., Caputo M.D., Salaroli A., Innocenti I., Frustaci A., Vitale C., Petullà M., De Fabritiis P., Vignetti M., Fazi P., Foà R.	Outcome of patients with re lapsed/refractory (R/R) chronic lymphocytic leukemia (CLL) and/or 17p deletion/*TP53* mutations treated with ibrutinib according to a named patient program (NPP) in Italy: Preliminary analysis of a real-life retrospective study	*Blood* (2016) 128:22. Date of Publication: 2 Dec 2016
Michallet A.-S., Campidelli A., Lequeu H., Dilhuydy M.-S., Tournilhac O., Fornecker L.-M., Dupuis J., Cymbalista F., De Guibert S., Delmer A., Vilque J.-P., Ghez D., Leblond V., Subtil F., Feugier P., Ysebaert L.	Ibrutinib in very elderly patients with relapsed/refractory chronic lymphocytic leukemia: A real-world experience of 71 patients treated in France: A study from the French Innovative Leukemia Organization (FILO) group	*American Journal of Hematology* (2017) 92:6 (E105-E107). Date of Publication: 1 Jun 2017
Morelli F., Tomasso A., Bacchiarri F., Gozzetti A., Laurenti L., Ciolli S.	CLL-251, Venetoclax for CLL Patients Outside Clinical Trials: An Italian Real-Life Experience	*Clinical Lymphoma, Myeloma and Leukemia* (2020) 20 Supplement 1 (S225-S226). Date of Publication: 1 Sep 2020
Nabhan C., Chung J., Mato A.R., Kish J., Nero D.	Comparison of costs and health care resource utilization (HRU) in chronic lymphocytic leukemia (CLL) patients treated with front-line ibrutinib or chemoimmunotherapy	*Blood* (2017) 130 Supplement 1. Date of Publication: 1 Dec 2017
Nadali G., Marchesini G., Facchinelli D., Farina F., Tisi M.C., Lessi F., Marchesi F., Cattaneo C., Candoni A., Fanci R., Prezioso L., Verga L., Laurenti L., Trastulli F., Picardi M., Del Principe M.I., Silva R., Busca A.	Infections in patients with lymphoproliferative diseases treated with target therapy. Italian multicentric retrospective study seifem 2017	*Blood* (2018) 132 Suppl. 1. Date of Publication: 1 Nov 2018
Nero D., Chung J., Kish J., Nabhan C.	Comparative study of healthcare resource utilization (HRU) outcomes between chronic lymphocytic leukemia (CLL) patients treated with ibrutinib versus non-ibrutinib treated patients	*Value in Health* (2017) 20:9 (A414). Date of Publication: 1 Oct 2017
Nero D., Chung J., Mato A.R., Kish J., Nabhan C.	Comparative study of cardiovascular co-morbidities between ibrutinib and non-ibrutinib-treated chronic lymphocytic leukemia (CLL) patients	*Blood* (2017) 130 Supplement 1. Date of Publication: 1 Dec 2017
Nohria A., Rosenblatt L., Pan X., Sharma A.	Evaluation of the risk of atrial fibrillation/flutter among patients initiating ibrutinib	*Blood* (2018) 132 Suppl. 1. Date of Publication: 1 Nov 2018
Olszewski A.J., Davids M.S., Yakirevich I., Egan P.C.	Early adoption and outcomes of ibrutinib as treatment for older patients with chronic lymphocytic leukemia (CLL): A population-based study	*Blood* (2019) 134 Supplement 1. Date of Publication: 1 Nov 2019
Panovská A., Němcová L., Nekvindová L., Špaček M., Šimkovič M., Papajík T., Brejcha M., Lysák D., Zuchnická J., Novák J., Starostka D., Poul H., Vrbacký F., Vodárek P., Urbanová R., Plevová K., Pospíšilová Š., Mašlejová S., Brychtová Y., Koriťáková E., Smolej L., Doubek M.	Real-world data on efficacy and safety of obinutuzumab plus chlorambucil, rituximab plus chlorambucil, and rituximab plus bendamustine in the frontline treatment of chronic lymphocytic leukemia: The GO-CLLEAR Study by the Czech CLL Study Group	*Hematological Oncology* (2020) 38:4 (509–516). Date of Publication: 1 Oct 2020
Puła B., Budziszewska B.K., Rybka J., Gil L., Subocz E., Długosz-Danecka M., Zawirska D., Waszczuk-Gajda A., Iskierka-Jażdżewska E., Kopacz A., Szymczyk A., Czyz J., Lech-Marańda E., Warzocha K., Jamroziak K.	Comparable efficacy of idelalisib plus rituximab and ibrutinib in relapsed/refractory chronic lymphocytic leukemia: A retrospective case matched study of the polish adult leukemia group (PALG)	*Anticancer Research* (2018) 38:5 (3025–3030). Date of Publication: 1 May 2018
Pula B., Iskierka-Jazdzewska E., Dlugosz-Danecka M., Szymczyk A., Hus M., Szeremet A., Drozd-Sokolowska J., Waszczuk-Gajda A., Zaucha J.M., Holojda J., Piszczek W., Steckiewicz P., Wojciechowska M., Osowiecki M., Knopinska-Posluszny W., Dudzinski M., Zawirska D., Subocz E., Halka J., Pluta A., Wichary R., Kumiega B., Budziszewska B.K., Jurczak W., Lech-Maranda E., Giannopoulos K., Robak T., Jamroziak K.	Long-term efficacy of ibrutinib in relapsed or refractory chronic lymphocytic leukemia: Results of the polish adult leukemia study group observational study	*Anticancer Research* (2020) 40:7 (4059–4066). Date of Publication: 1 Jul 2020
Puła B., Iskierka-Jazdzewska E., Długosz-Danecka M., Szymczyk A., Hus M., Szeremet A., Drozd-Sokołowska J., Waszczuk-Gajda A., Zaucha J.M., Hołojda J., Piszczek W., Steckiewicz P., Wojciechowska M., Osowiecki M., Knopińska-Posłuszny W., Dudziński M., Zawirska D., Subocz E., Hałka J., Pluta A., Wichary R., Kumiega B., Budziszewska B.K., Jurczak W., Lech-Marańda E., Giannopoulos K., Robak T., Jamroziak K.	Long-term real-world clinical outcomes for ibrutinib monotherapy treatment in relapsed refractory chronic lymphocytic leukemia-observational study of the polish adult leukemia study group (PALG)	*HemaSphere* (2020) 4 Supplement 1 (867–868). Date of Publication: 1 Jun 2020
Puła B., Iskierka-Jazdzewska E., Długosz-Danecka M., Szymczyk A., Hus M., Szeremet A., Rybka J., Drozd-Sokołowska J., Waszczuk-Gajda A., Zaucha J.M., Hołojda J., Piszczek W., Steckiewicz P., Wojciechowska M., Osowiecki M., Knopińska-Posłuszny W., Kopacz A., Dudziński M., Zawirska D., Piotrowska M., Subocz E., Hałka J., Kumiega B., Gil L., Szukalski L., Wichary R., Budziszewska B.K., Lech-Maranda E., Jurczak W., Giannopoulos K., Robak T., Warzocha K., Jamroziak K.	Second cancers in chronic lymphocytic leukemia patients treated with BCR inhibitors:-Retrospective analysis of the polish adult leukemia study group (PALG)	*HemaSphere* (2018) 2 Supplement 2 (851–852). Date of Publication: 1 Jun 2018
Rotbain E.C., Frederiksen H., Hjalgrim H., Rostgaard K., Egholm G.J., Zahedi B., Poulsen C.B., Enggard L., Da Cunha-Bang C., Niemann C.U.	IGHV mutational status and outcome for patients with chronic lymphocytic leukemia upon treatment: A danish nationwide population-based study	*Haematologica* (2020) 105:6 (1621–1629). Date of Publication: 1 Jun 2020
Ryan K., Burudpakdee C., Zhao X., Le H., Near A.	Characteristics of mantle cell lymphoma (MCL) and chronic lymphocytic leukemia (CLL) patients treated with acalabrutinib in a real world setting in the United States	*Blood* (2019) 134 Supplement 1. Date of Publication: 1 Nov 2019
Sabrie N., Leong D., Prica A., Austin P., Pang A., Fang J., Calvillo-Arguelles O., Lee D., Thavendiranathan P., Abdel-Qadir H.	INCREASED RISK OF ADVERSE CARDIOVASCULAR EVENTS ASSOCIATED WITH IBRUTINIB USE IN CHRONIC LYMPHOCYTIC LEUKEMIA: A PROPENSITY-MATCHED POPULATION-BASED COHORT STUDY	*Journal of the American College of Cardiology* (2020) 75:11 (414). Date of Publication: 24 Mar 2020
Sandoval-Sus J.D., Chavez J.C., Dalia S., Bello C.M., Shah B.D., Ho V.Q., Nodzon L., Kharfan-Dabaja M.A., Sotomayor E.M., Sokol L., Pinilla-Ibarz J.	Outcomes of patients with relapsed/refractory chronic lymphocytic leukemia after ibrutinib discontinuation outside clinical trials: A single institution experience	*Blood* (2015) 126:23 (2945). Date of Publication: 3 Dec 2015
Scalzulli P.R., Guarini A., Loseto G., Specchia G., Giordano A.M., Pastore D., Quintana G., Mazza P., Maggi A., Di Renzo N., De Paolis M.R., Tarantini G., De Santis G., Pavone V., Greco A., Valvano M.R., Cascavilla N.	Ibrutinib, single agent BTK inhibitor, for treatment of Naïve (TN) and relapsed/refractory (R/R) chronic lymphocytic leukemia: A real-life experience from rete ematologica pugliese (REP)	*Blood* (2018) 132 Suppl. 1. Date of Publication: 1 Nov 2018
Schetelig J., Chevallier P., van Gelder M., Hoek J., Hermine O., Chakraverty R., Browne P., Milpied N., Malagola M., Socié G., Delgado J., Deconinck E., Damaj G., Maury S., Beelen D., Quoc S.N., Shankara P., Brecht A., Mayer J., Hunault-Berger M., Bittenbring J., Thieblemont C., Lepretre S., Baldauf H., de Wreede L.C., Tournilhac O., Yakoub-Agha I., Kröger N., Dreger P.	Idelalisib treatment prior to allogeneic stem cell transplantation for patients with chronic lymphocytic leukemia: a report from the EBMT chronic malignancies working party	*Bone Marrow Transplantation* (2020). Date of Publication: 2020
Seymour E.K., Ruterbusch J.J., Beebe-Dimmer J.L., Schiffer C.A.	Real-world testing and treatment patterns in chronic lymphocytic leukemia: A SEER patterns of care analysis	*Cancer* (2019) 125:1 (135–143). Date of Publication: 1 Jan 2019
Shadman M., Sail K., Manzoor B.S., Yazdy M.S., Hill B.T., Tuncer H.H., Allan J.N., Ujjani C.S., Emechebe N., Kamalakar R., Sharmokh S., Jiang D.D., Pena G., Marshall T., Nielsen J., Barr P.M., Brown J.R., Schuh A., Eyre T.A., Lamanna N., Wierda W.G., Skarbnik A., Roeker L.E., Bannerji R., Pauff J.M., Schuster S.J., Follows G.A., Cheson B.D., Eichhorst B.F., Brander D.M., Pivneva I., Guerin A., Mato A.R.	Treatment discontinuation patterns for patients with CLL in the real-world settings: Results from a multi-center study	*Blood* (2019) 134 Supplement 1. Date of Publication: 1 Nov 2019
Sharman J.P., Black-Shinn J.L., Clark J., Bitman B.	Understanding ibrutinib treatment discontinuation patterns for chronic lymphocytic leukemia	*Blood* (2017) 130 Supplement 1. Date of Publication: 1 Dec 2017
Silva S., Espada E., Melo J.A., Lima M.P., Ionita A., Carda J.P., Andrade J., Neves M., Cabral R., Mendes T., Gaspar C., Alves D., Pina F., Botelho De Sousa A., Coelho H., Montalvão A., Vitória H., Lima F., Coutinho J., Lúcio P., Guimarães J.E., Ribeiro M.L., Gomes Da Silva M., Raposo J.	Portuguese real-life experience with ibrutinib outside clinical trials: - A multicenter analysis	*Hematological Oncology* (2017) 35 Supplement 2 (383–384). Date of Publication: 1 Jun 2017
Sylvan S.E., Asklid A., Johansson H., Klintman J., Bjellvi J., Tolvgård S., Kimby E., Norin S., Andersson P.-O., Karlsson C., Karlsson K., Lauri B., Mattsson M., Sandstedt A.B., Strandberg M., Österborg A., Hansson L.	First-line therapy in chronic lymphocytic leukemia: A swedish nation-wide real-world study on 1053 consecutive patients treated between 2007 and 2013	*Haematologica* (2019) 104:4 (797–804). Date of Publication: 31 Mar 2019
Tam C., Kuss B., Opat S., Puig A., Acar M., Zhou C., Mulligan S.	Real world treatment persistence of australian ibrutinib patients in a named patient program	*HemaSphere* (2018) 2 Supplement 2 (129–130). Date of Publication: 1 Jun 2018
Tejaswi V., Lad D.P., Jindal N., Prakash G., Malhotra P., Khadwal A., Jain A., Sreedharanunni S., Singh Sachdeva M., Naseem S., Varma N., Varma S.	Chronic Lymphocytic Leukemia: Real-World Data from India	*JCO Global Oncology* (2020):6 (866–872). Date of Publication: 24 Jun 2020
Tombak A., Tanrikulu F.P., Durusoy S.S., Gurkan E., Kaya E., Umit E.G., Yavasoglu I., Mehtap Ö., Deveci B., Ozcan M.A., Terzi H., Okay M., Sayinalp N., Yilmaz M., Okan V., Kizikli A., Ozcan O., Çetin G., Demircioglu S., Aydogdu I., Saydam G., Davulcu E.A., Ilhan G., Ucar M.A., Ozet G., Akpinar S., Turgut B., Berber I., Kurtoglu E., Sönmez M., Batur D.S., Yildirim R., Ozkocaman V., Gunes A.K., Sahip B., Ertop S., Akay M., Basturk A., Dogu M.H., Akdeniz A., Unal A., Seyhanli A., Ferhanoglu B.	Efficacy and safety of ibrutinib use in patients with chronic lymphocytic leukemia in real-world experiences: Results of a prospective multicenter study	*Blood* (2019) 134 Supplement 1. Date of Publication: 1 Nov 2019
Uminski K., Brown K., Bucher O., Hibbert I., Dhaliwal D.H., Johnston J.B., Geirnaert M., Dawe D.E., Banerji V.	Descriptive analysis of dosing and outcomes for patients with ibrutinib-treated relapsed or refractory chronic lymphocytic leukemia in a Canadian center	*Current Oncology* (2019) 26:5 (e610-e617). Date of Publication: 2019
van der Straten L., Kater A.P., Doorduijn J.K., van den Broek E.C., Posthuma E.F.M., Dinmohamed A.G., Levin M.-D.	Possible hampered effectiveness of second-line treatment with rituximab-containing chemotherapy without signs of rituximab resistance: a population-based study among patients with chronic lymphocytic leukemia	*Annals of Hematology* (2020) 99:5 (1081–1091). Date of Publication: 1 May 2020
Van Der Straten L., Levin M.-D., Visser O., Blijlevens N.M., Cornelissen J.J., Doorduijn J.K., Kater A.P., Dinmohamed A.G.	Effectiveness of ibrutinib for the treatment of chronic lymphocytic leukemia in daily practice in the netherlands: A nationwide population-based cohort study	*HemaSphere* (2018) 2 Supplement 2 (131). Date of Publication: 1 Jun 2018
Vanderveer E., Huang S.J.T., Bruyere H., Gillan T., Li C.H., Ramadan K., Villa D., Scott D.W., Savage K.J., Connors J.M., Sehn L.H., Toze C.L., Gerrie A.S.	Oral fludarabine and intravenous rituximab (FR) for chronic lymphocytic leukemia (CLL): Long term outcomes and secondary malignancies in 673 patients treated in British Columbia (BC)	*Blood* (2019) 134 Supplement 1. Date of Publication: 1 Nov 2019
Vitale C., Salvetti C., Griggio V., Scamuffa M.C., Zamprogna G., Visentin A., Cassin R., Laurenti L., Murru R., Rivela P., Marchetti M., Gentile M., Pennese E., Reda G., Trentin L., Tedeschi A., Mauro F.R., Foà R., Boccadoro M., Coscia M.	Pre-existing and treatment-emergent autoimmune cytopenias in patients with chronic lymphocytic leukemia treated with targeted drugs	*Blood* (2019) 134 Supplement 1. Date of Publication: 1 Nov 2019
Wang S., Emond B., Romdhani H., Lefebvre P., Sundaram M., Mato A.	Ibrutinib treatment is associated with lower healthcare resource utilization and net cost savings compared to chemoimmunotherapy in front-line cll oncology care model episodes	*Journal of Managed Care and Specialty Pharmacy* (2018) 24:10 A (S28-S29). Date of Publication: 1 Oct 2018
Wang S., Emond B., Romdhani H., Lefebvre P., Sundaram M., Mato A.R.	Front-line ibrutinib treatment is associated with longer time to next treatment, net total cost reduction, and lower healthcare resource utilization compared to chemoimmunotherapy in patients with chronic lymphocytic leukemia	*Blood* (2018) 132 Suppl. 1. Date of Publication: 1 Nov 2018
Weinkove R., Doocey R., Henderson R., Islam S., Smith M., Puig A., Pateman G., Acar M., Amaya-Chanaga C., Simpson D.	Real world treatment persistence of New Zealand ibrutinib chronic lymphocytic leukemia patients in a named patient program	*HemaSphere* (2019) 3 Supplement 1 (861–862). Date of Publication: 1 Jun 2019
Winqvist M., Andersson P.-O., Asklid A., Karlsson K., Karlsson C., Lauri B., Lundin J., Mattsson M., Norin S., Sandstedt A., Rosenquist R., Späth F., Hansson L., Österborg A.	Long-term real-world results of ibrutinib therapy in patients with relapsed or refractory chronic lymphocytic leukemia: A 30-month follow up of the swedish compassionate use cohort	*Haematologica* (2019) 104:5 (e208-e210). Date of Publication: 30 Apr 2019
Winqvist M., Andersson P.-O., Asklid A., Karlsson K., Karlsson C., Lauri B., Lundin J., Mattsson M., Norin S., Sandstedt A.B., Hansson L., Österborg A.	Real-world results on ibrutinib in relapsed/refractory CLL: A 21-month follow-up of 95 swedish patients treated in a compassionate use program	*Blood* (2017) 130 Supplement 1. Date of Publication: 1 Dec 2017
Yazdy M.S., Mato A.R., Roeker L.E., Jarral U., Ujjani C.S., Shadman M., Skarbnik A., Whitaker K.J., Deonarine I., Kabel C.C., Stump S.E., Goodfriend J., Pagel J.M., Bailey N., Patel K., Jacobs R., Feldman T.A., Leslie L.A., Goy A., Coombs C.C., Muluneh B., Khajavian S., Lamanna N., Weissbrot H., Weiss J., Cheson B.D.	Toxicities and outcomes of acalabrutinib-treated patients with chronic lymphocytic leukemia: A retrospective analysis of real-world patients	*Blood* (2019) 134 Supplement 1. Date of Publication: 1 Nov 2019
Ysebaert L., Aurran-Schleinitz T., Dartigeas C., Dilhuydy M.S., Feugier P., Michallet A.S., Tournilhac O., Dupuis J., Sinet P., Albrecht C., Cymbalista F.	Large scale, real-world results on ibrutinib for 428 french patients with 17p deletion or relapsed/refractory chronic lymphocytic leukemia included in a temporary authorization for use in the (ATU) program	*Haematologica* (2016) 101 Supplement 1 (57–58). Date of Publication: 1 Jun 2016
Zaja F., Mian M., Volpetti S., Visco C., Sissa C., Nichele I., Castelli M., Ambrosetti A., Puglisi S., Fanin R., Cortelazzo S., Pizzolo G., Trentin L., Rodeghiero F., Paolini R., Vivaldi P., Sancetta R., Isola M., Semenzato G.	Bendamustine in chronic lymphocytic leukemia: Outcome according to different clinical and biological prognostic factors in the everyday clinical practice	*American Journal of Hematology* (2013) 88:11 (955–960). Date of Publication: November 2013

**Table A2:** Added references.

Authors	Title	Source
Gentile M., et al.	Validation of a survival-risk score (SRS) in relapsed/refractory CLL patients treated with idelalisib–rituximab	*Blood Cancer Journal* (2020) 10:9 Article Number 92
Morelli F., et al.	Treatment of chronic lymphocytic leukemia in the new drugs era: The state of art in the Italian landscape	*HemaSphere* (2020) 4 Supplement 1 (315–316)
Ysebaert L., et al.	Non-interventional retrospective multicenter study evaluating real-word idelalisib use in CLL and INHL patients enrolled in the French early access program (EAP)	*HemaSphere* (2019) 3 Supplement 1 (862–863)
Salles G, et al.	Single-agent ibrutinib in RESONATE-2™ and RESONATE™ versus treatments in the real-world PHEDRA databases for patients with chronic lymphocytic leukemia	*Annals Hematology* (2019) Dec; 98(12): 2749–2760.
Azali L, et al.	Evaluation of the incidence and risk factors associated with major cardiovascular events in patients receivingacalabrutinib therapy	*ASH* (2020) Abstract 2223
Farrukh T. Awan, et al.	Acalabrutinib monotherapy in patients with chronic lymphocytic leukemia who are intolerant to ibrutinib	*Blood Advances* (2019) 3:1553–62
Innocenti et al.	Venetoclax in CLL patients who progress after B-cell Receptor inhibitor treatment: A retrospective multicenter Italian experience	*British Journal of Haematology* (2019) Oct; 187(1): e8–e11.
Alsina et al.	Role of age, fitness and concomitant medications in CLL patients treated with venetoclax	Abstract ASH (2020) Blood, *Blood* (2020) 136 (Supplement 1): 25–26.
